# What Is New on Ovarian Carcinoma: Integrated Morphologic and Molecular Analysis Following the New 2020 World Health Organization Classification of Female Genital Tumors

**DOI:** 10.3390/diagnostics11040697

**Published:** 2021-04-14

**Authors:** Antonio De Leo, Donatella Santini, Claudio Ceccarelli, Giacomo Santandrea, Andrea Palicelli, Giorgia Acquaviva, Federico Chiarucci, Francesca Rosini, Gloria Ravegnini, Annalisa Pession, Daniela Turchetti, Claudio Zamagni, Anna Myriam Perrone, Pierandrea De Iaco, Giovanni Tallini, Dario de Biase

**Affiliations:** 1 Department of Experimental, Diagnostic and Specialty Medicine, Alma Mater Studiorum—University of Bologna, Via Massarenti 9, 40138 Bologna, Italy; claudio.ceccarelli@unibo.it (C.C.); giorgia.acquaviva3@unibo.it (G.A.); federico.chiarucci2@studio.unibo.it (F.C.); giovanni.tallini@unibo.it (G.T.); 2 Molecular Pathology Laboratory, IRCCS Azienda Ospedaliero—Universitaria di Bologna/Azienda USL di Bologna, 40138 Bologna, Italy; annalisa.pession@ausl.bologna.it (A.P.); dario.debiase@unibo.it (D.d.B.); 3 Centro di Studio e Ricerca delle Neoplasie Ginecologiche, Alma Mater Studiorum—University of Bologna, 40138 Bologna, Italy; donatella.santini@aosp.bo.it (D.S.); gloria.ravegnini2@unibo.it (G.R.); daniela.turchetti@unibo.it (D.T.); myriam.perrone@aosp.bo.it (A.M.P.); pierandrea.deiaco@unibo.it (P.D.I.); 4 Pathology Unit, IRCCS Azienda Ospedaliero—Universitaria di Bologna, Via Massarenti 9, 40138 Bologna, Italy; francesca.rosini@aosp.bo.it; 5 Pathology Unit, AUSL-IRCCS di Reggio Emilia, 42122 Reggio Emilia, Italy; giacomo.santandrea@ausl.re.it (G.S.); andrea.palicelli@ausl.re.it (A.P.); 6 Department of Pharmacy and Biotechnology, University of Bologna, 40126 Bologna, Italy; 7 Unit of Medical Genetics, IRCCS Azienda Ospedaliero—Universitaria di Bologna, Via Massarenti 9, 40138 Bologna, Italy; 8 IRCCS Azienda Ospedaliero—Universitaria di Bologna, Via Albertoni 15, 40138 Bologna, Italy; claudio.zamagni@aosp.bo.it; 9 Division of Gynecologic Oncology, IRCCS Azienda Ospedaliero—Universitaria di Bologna, Via Massarenti 13, 40138 Bologna, Italy

**Keywords:** ovarian carcinoma, histopathology, immunohistochemical profile, molecular pathology, ovarian cancer tissue biomarkers

## Abstract

Ovarian carcinomas represent a heterogeneous group of neoplasms consisting of separate entities with distinct risk factors, precursor lesions, pathogenesis, patterns of spread, molecular profiles, clinical course, response to chemotherapy, and outcomes. The histologic subtype and the related molecular features are essential for individualized clinical decision-making. The fifth edition of the World Health Organization classification of tumors of the female genital tract divides ovarian carcinomas into at least five main and distinct types of ovarian carcinomas: high-grade serous carcinoma, low-grade serous carcinoma, endometrioid carcinoma, clear cell carcinoma, and mucinous carcinoma. Molecular pathology has improved the knowledge of genomic landscape of ovarian carcinomas identifying peculiar alterations for every histologic subtype. It is well-known that high-grade and low-grade serous carcinomas are separate entities with entirely different morphologic and molecular characteristics. *TP53* and *BRCA* mutations are typical of high-grade serous carcinoma, whereas *BRAF* and *KRAS* mutations frequently occur in low-grade serous carcinoma. Endometrioid and clear cell carcinomas are frequently associated with endometriosis. Endometrioid tumors are characterized by β-catenin alterations, microsatellite instability, and *PTEN* and *POLE* mutations, while *ARID1A* mutations occur in both endometrioid and clear cell carcinomas. Mucinous carcinomas are uncommon tumors associated with copy-number loss of *CDKN2A* and *KRAS* alterations and metastasis from other sites should always be considered in the differential diagnosis.

## 1. Introduction

Ovarian cancer represents the second most common malignant gynecologic neoplasm, in Western countries and it is accounted for more mortality than all other female genital tumors [1]. Ovarian tumors are divided in epithelial, representing almost 90% of cases, germ cell (3%), and sex cord-stromal (2%) [2]. It is worth noting that the current gynecologic cancer treatment differs from the past management. Malignant epithelial ovarian cancer (carcinoma) was traditionally considered to be a single disease, with treatment approaches based principally on grade and stage, but it is now evident that the histologic and molecular subtyping is essential for patient management. In fact, ovarian carcinoma represents a heterogenous disease consisting of a group of tumors, each with different precursor lesions, pathogenesis, patterns of spread, response to chemotherapy, and prognosis [3,4]. Recent studies have allowed to deepen the biology, the molecular alterations, the different sites of origin of ovarian carcinomas, opening the opportunity for a more personalized therapeutic approach and treatments with targeted drugs (e.g., PARP inhibitors) [5,6,7]. In particular, massive molecular characterization studies have improved the comprehension of ovarian carcinomas genomics identifying peculiar alterations for each histologic subtype. Despite the attractive and consistent advances from molecular pathology to treatment, the fifth edition of the WHO classification of female genital tumors maintains most of the diagnostic entities of the previous edition, enriching them with new histopathological, immunohistochemical, and molecular data. According to the new 2020 World Health Organization classification, at least five main types of ovarian carcinomas are identified based on histopathology, immunoprofile, and molecular analysis: high-grade serous carcinoma (HGSC, 70%), endometrioid carcinoma (EC, 10%), clear cell carcinoma (CCC, 6–10%), low-grade serous carcinoma (LGSC, 5%), and mucinous carcinoma (MC, 3–4%) [2,4]. Some rare entities have been introduced (e.g., mesonephric-like carcinoma and mixed carcinoma), while others have been removed (e.g., seromucinous carcinoma).

The most important novelty of the fifth edition of the WHO classification of female genital tumors concerns the integration of modern diagnostic criteria with immuno-molecular algorithms for a better definition and highly diagnostic reproducibility of the different main histotypes (Table 1).

Serous carcinomas: as in the previous classification, serous carcinomas are divided into LGSC and HGSC that represent two separate tumor types with different morphology, pathogenesis, molecular events, and prognosis. HGSCs are associated with *TP53* mutations (more than 97%) and homologous recombination deficiency (HRD) including *BRCA* mutations, while LGSCs are characterized by *BRAF* or *KRAS* mutations. It is now well-known that the vast majority of so-called ovarian HGSC arises from the distal fimbrial end of the fallopian tube from a precursor lesion known as serous tubal intraepithelial carcinoma (STIC), whereas almost all LGSCs arise within the ovary from benign and borderline serous tumors. The WHO provides criteria for site assignment in extrauterine HGSCs and the use of these criteria leads to classification of a high percentage of cases (~80%) as being of tubal origin, whereas primary peritoneal HGSCs are extremely rare.

Endometrioid carcinomas: the innovative and biologically informative molecular classification of endometrial carcinoma provided by The Cancer Genome Atlas (TCGA) has been translated and applied in the characterization of molecular subtypes of ovarian endometrioid carcinoma: ultramutated due to *POLE* exonuclease domain mutations (~5%), hypermutated due to mismatch repair deficiency (MMRd; ~13%), *TP53*-mutated (9–13%), and no specific molecular profile (NSMP; 69–73%). Furthermore, seromucinous carcinoma is now considered a subtype of endometrioid carcinoma for its analogous molecular features.

Clear cell carcinoma: most of tumors arise from transformed ovarian endometriosis or benign and borderline tumors. About 40–50% of cases harbor loss-of-function mutations in ARID1A, PIK3CA mutations are common, while KRAS mutations (10%), TP53 mutations (<10%), and mismatch repair deficiency (0–6%) are uncommon.

Mucinous carcinoma: uncommon tumors associated with copy-number loss of CDKN2A (76%), KRAS and TP53 mutations (both 64%), and ERBB2 (HER2) amplifications (15–26%).

This review aims to highlight the clinicopathologic and molecular characteristics of ovarian carcinomas focusing on histologic features, immunohistochemical profile, and molecular tissue biomarkers.

## 2. High-Grade Serous Carcinoma

High-grade serous carcinoma (HGSC) represents the most common ovarian cancer accounting for about 70% of ovarian carcinoma [2]. The median age of patients is 56 years (ranging from 45 to 65 years). Currently, screening techniques are unsuccessful for early detection of cancer and, therefore, most patients (~80%) present with advanced-stage disease; tumors limited to the ovary at presentation are uncommon (<5%). Typically, HGSC presents at diagnosis with bilateral ovarian involvement, diffuse and extensive peritoneal carcinosis particularly with omental involvement [3,8]. Advanced intra-abdominal tumor is often associated with signs of intestinal obstruction, including nausea, vomiting, persistent bloating, and abdominal pain. Ultrasound, MRI, and CT have no clearly defined role in preoperative tumor staging. Laparotomy and surgical exploration of the abdominal cavity remain the standard approach for staging.

Women with deleterious germline *BRCA1* or *BRCA2* mutations have a 30–70% risk of developing HGSC by age of 70 [9,10].

### 2.1. Pathology of High-Grade Serous Carcinoma

**Figure 1:**
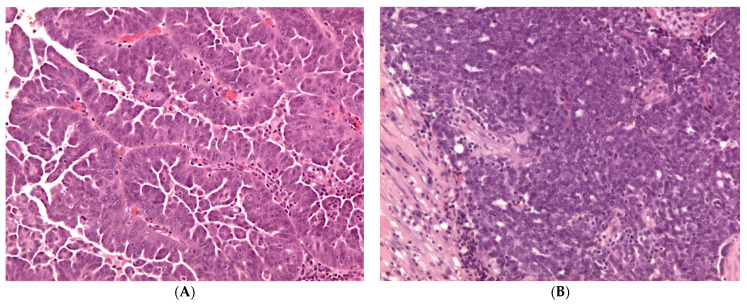
(**A**) Histologic appearance of classic high-grade serous carcinoma (HGSC) (hematoxylin and eosin, H&E, ×100); (**B**) Solid, pseudoendometrioid, transitional cell carcinoma-like (SET) variant of HGSC (hematoxylin and eosin, H&E, ×100).

Microscopically, HGSCs are heterogeneous and recently two histological types have been described: the classic type and SET (Solid, pseudoEndometrioid and Transitional) variant [2,11]. Classic HGSC shows variable architectural features including papillary, micropapillary and solid growth patterns. The tumor cells typically exhibit marked nuclear pleomorphism with prominent nucleoli, high mitotic activity (typically > 12 mitoses per 10 high-power fields), including atypical mitoses. SET variant is characterized by solid sheets of cells simulating endometrioid and/or transitional cell carcinomas. Micropapillae and bizarre giant cells may also be seen. These tumors often show geographical necrosis and are associated with high count of tumor-infiltrating lymphocytes (TILs) (Figure 1) [11].

Classic and SET tumors have an identical immunoprofile characterized by positivity for Wilms tumor 1 (WT1), p16, mutation-type pattern of p53, and variable expression of Estrogen and Progesterone receptors (ER and PR). HGSC abnormal pattern of p53 expression (mutation-type labelling) is represented by strong diffuse staining in at least 80% of cells (overexpression), no expression (null), or (rarely) diffuse cytoplasmic staining with weak nuclear staining [12,13]. Soslow and collaborators have shown that SET pattern of HGSC has been more commonly associated with germline and/or somatic *BRCA1/2* mutations [11,14]. In fact, *BRCA*-associated HGSC had frequent SET features, higher mitotic activity, more TILs, and either geographic or comedo necrosis. Algorithms incorporating tumor architecture, necrosis, mitotic index, and TILs count may separate *BRCA*-associated carcinomas from those *BRCA* unassociated. These data indicated potential strong associations between morphology and genotype in HGSC. In addition, some studies have evaluated the prognostic impact of the higher-level T cell infiltration and HGC morphology [15]. Furthermore, as will be discussed later, the morphological subtypes correlate with different sensitivity to chemotherapy and PARP inhibitors.

The traditional hypothesis of HGSC origin from ovarian surface epithelium or cortical inclusion cysts has been revolutionized by molecular and morphologic evidences that the majority of ovarian (~80%) HGSCs derives from the epithelium of the fimbria of the fallopian tube [16,17,18,19,20,21,22]. In fact, the traditional view has been changed by the identification of serous tubal intraepithelial carcinoma (STIC) in the fallopian tubes of prophylactic salpingo-oophorectomies carried out for women with *BRCA1* or *BRCA2* germline mutations. Like HGSC, STIC shows diffuse and strong expression of p53, and the Ki-67 proliferation index is usually greater than 10% [18,23,24,25,26,27,28,29]. The identification of identical *TP53* mutations in both STIC and concordant ovarian HGSC has highlighted the clonal relationship between them supporting that the fallopian fimbria is the site of origin of most HGSCs. Furthermore, some studies have been demonstrated that STIC is not a precursor lesion but represents the early histologically detectable form of HGSC and it can disseminate to the ovary and metastasize [18,21,30,31]. In order to identify early cancer in risk-reducing salpingo-oophorectomy (RRSO) specimens of *BRCA* patients, a pathologic protocol for Sectioning and Extensively Examining the Fimbriated End (SEE-FIM protocol) has been developed [23,24,32,33]. According to this method, the tube is extensive sectioned and entirely submitted for histologic evaluation. The routine application of SEE-FIM protocol resulted in detection of several types of pathologic lesions ranging from the so-called “p53-signature—normal-appearing tubal epithelium that overexpresses p53—to lesions displaying cytologic atypia that falls short of STIC, referred to as “serous tubal intraepithelial lesion” (STIL). Like STIC, p53 signature and STIL are composed of at least 12 consecutive secretory cells that show strong and diffuse immunoexpression of p53 [28,29,32]. Both precursor lesions contain *TP53* mutations with low Ki-67 proliferation index (mean, 3%) and are more frequently identified in association with STIC. This evidence suggested that tubal lesions with *TP53* mutation are an initial event in HGSC pathogenesis. In addition, another tubal alteration consisting of linear expansion of secretory cells has been recently described as SCOUT (Secretory Cell OUTgrowth) defined as secretory cell outgrowth to 30 or more cells with alterations in gene function analogous to p53 signatures. In summary, the most recent hypothesis of HGSC pathogenesis provides a stepwise progression of the tubal epithelium to precursor lesions to carcinoma, with the sequence “SCOUT-p53 signature-STIL-STIC-HGSC” [25,27,28,34]. In fact, tubal secretory cells seem to have a limited ability to repair DNA damage and to be especially sensitive to *BRCA* mutations. However, all the recent advances in understanding the pathogenesis of HGSC highlight how it appears to be more complex: HGSC includes a heterogeneous group of diseases [34,35,36].

Several studies have detected STIC in only 40% of advanced HGSC. Furthermore, paradoxically, it has been observed a negative correlation between SET features and coexisting STIC in suggesting that the differences in morphology may be correlated to different pathways of tumor evolution. Some authors have proposed a dualistic model of HGSC based on several variables including age, *BRCA* mutation status, histology (classic versus SET), STIC, and patient outcome—identifying two tumor groups: (I) younger *BRCA*-mutated patients, with SET morphology, STIC-negative, more responsive to chemotherapy and PARP inhibitors, with a favorable outcome; and (II) older patients without *BRCA* alterations, with classic morphology, STIC-positive, less responsive to chemotherapy, with an unfavorable prognosis.

The WHO classification emphasizes the importance of defining the site of origin of HGSCs using defined criteria (Table 2) [37]. Application of these criteria for site assignment in HGSCs leads to classification of a high percentage of cases (~80%) as being of tubal origin, whereas primary peritoneal HGSCs are extremely rare. Peritoneal origin should be considered only after exclusion of the presence of tubal STIC or HGSC and absence of ovarian involvement.

### 2.2. Molecular Features of High-Grade Serous Carcinoma

The mutational spectrum distinguishes HGSC as completely separate from other histological subtypes of ovarian carcinoma reflecting a combination of etiological and oncogenetic characteristics. The Cancer Genome Atlas (TCGA) project provided a comprehensive integrative profile of the aberrations in HGSC using microarrays and massively parallel sequencing [38]. The TCGA project has analyzed mRNA expression, miRNA expression, promoter methylation, and DNA copy-number in 489 high-grade serous carcinomas and the DNA sequences of exons from coding genes in 316 of these tumors. Interestingly, the mutational analysis identified *TP53* mutations in almost all tumors (96%). *BRCA1* and *BRCA2* germline and somatic mutations were found to be mutated in 22% of cancers. In addition to *TP53* and *BRCA1/2* mutations, other molecular characteristics included somatic copy-number alterations (SCNAs), which is correlated to genomic instability, *CCNE1* amplification, and promoter methylation of 168 genes. Homologous recombination deficiency (HRD) due to genetic or epigenetic inactivation of DNA damage repair genes, such as *BRCA1/2*, has been found in about 50% of HGSCs [38,39]. Massive sequencing techniques identified mutations in non-*BRCA* HR genes including *ATM*, *BARD1*, *BRIP1*, *CHEK1*, *CHEK2*, *FAM175A*, *MRE11A*, *NBN*, *PALB2*, *RAD51C*, and *RAR51D* [40]. The main challenge is the interpretation of the pathogenic significance of some variants and their clinical relevance [41]. While some mutations are pathogenic because they produce abnormal proteins, others have an unknown effect because they do not result in significant changes in proteins.

Gene expression analysis of HGSC identified four molecular subtypes that were designated as “immunoreactive”, “proliferative”, “differentiated”, and “mesenchymal” [38,39]. These molecular subtypes were associated with distinct clinical outcomes: the immunoreactive subtype associated with *BRCA1* disruptions and high TILs had a better prognosis. It is postulated that these subtypes may reflect distinct patterns of oncogene activation. Several molecular studies suggest that high-grade serous carcinogenesis is initiated by early p53 loss followed by *BRCA* loss, leading to disruption of DNA repair, followed by chromosomal instability and copy-number alterations that represent the major determinant of progression of HGSC. The findings of the TCGA project support this model of carcinogenesis where the most characteristic abnormalities are *TP53* mutations and widespread DNA copy-number alterations [36,40,42].

## 3. Low-Grade Serous Carcinoma

Low-grade serous carcinomas account for approximately 3% of all ovarian carcinomas and are advanced-stage at diagnosis [2]. Patients present over a wide age range (median: 43 years), approximately a decade younger than those with HGSC. Most of LGSCs (about 50%) are associated with a serous borderline tumor (SBT) component (with or without micropapillary architecture) from which they are presumed to derive. Progression of SBT into LGSC occurs only in 6–7% of patients and evolution to HGSC occurs rarely. Although LGSC has relatively indolent growth, advanced-disease is correlated with a worse prognosis because poorly responsive to conventional platinum-based chemotherapy [43].

### 3.1. Pathology of Low-Grade Serous Carcinoma

**Figure 2:**
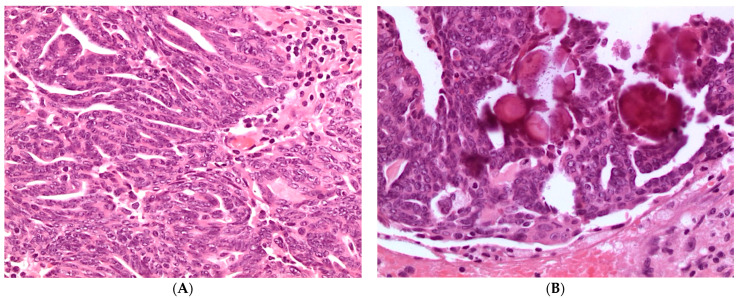
(**A**) Histologic appearance of low-grade serous carcinoma showing tumor cells with uniform nuclei and inconspicuous mitotic activity (hematoxylin and eosin, H&E, ×100); (**B**) Psammoma bodies are frequent in low-grade serous carcinoma (hematoxylin and eosin, H&E, ×100).

Microscopically, LGSC is composed of homogeneous population of small cells with scant cytoplasm arranged in small papillae. In contrast to HGSC, tumor cells show mild to moderate nuclear atypia, without pleomorphism (<3× variation in size) and may have prominent nucleoli. Mitoses are usually less than 12 per 10 high-power fields. Psammoma bodies are frequent. LGSC is differentiated from SBT by the presence of stromal invasion greater than microinvasion (invasive foci measuring > 5 mm or 10 mm^2^) (Figure 2).

Low-grade serous carcinomas typically express WT1, CK7, PAX8, Estrogen receptor (ER), and Progesterone receptor (PR). Unlike HGSC, the Ki-67 proliferation index is low (usually less than 3%) and p53 shows a wild-type expression [13].

### 3.2. Molecular Features of Low-Grade Serous Carcinoma

LGSC is not associated with *BRCA* germline mutations and does not show chromosomal instability seen in HGSCs. The most common molecular alterations include mutations of *KRAS*, *NRAS*, *BRAF*, *USP9X*, *EIF1AX*, and *ERBB2* genes. *BRAF* or *KRAS* mutations occur in LGSCs in 30% and 35%, respectively, while *ERBB2* mutation is uncommon (less than 5% of tumors). These alterations appear to be early events in LGSC pathogenesis as they have also been identified in benign cystadenomas and SBTs associated with carcinoma. In addition, some authors have reported a better prognosis for LGSCs *BRAF* mutated than for those with *KRAS* mutations or with *BRAF* and *KRAS* wild-type. Furthermore, the identification of *KRAS* alterations in peritoneal SBT implants has been associated with a worse outcome suggesting that such mutations could be used as a biomarker for risk assessment in this subset of patients [44]. Interestingly, a recent comprehensive genomic study identified 47% of cases with mutations in key *RAS*/*RAF* pathway genes (*KRAS*, *BRAF*, and *NRAS*), as well as mutations in putative novel driver genes including *USP9X* (27%), *MACF1* (11%), *ARID1A* (9%), *NF2* (4%), *DOT1L* (6%), and *ASH1L* (4%) [44].

## 4. Endometrioid Carcinoma

Endometrioid carcinoma (EC) accounts for approximately 10–15% of all ovarian carcinomas representing the second most common histotype [2]. In recent years, the incidence of ovarian endometrioid carcinoma, seems to have been decreased as many of the “high-grade endometrioid carcinomas” have recently been reclassified as SET variants of HGSC. The median age of patients is 51 years (ranging from 26 to 87 years). Most ECs are found at an early stage (FIGO stage I or II) at diagnosis. The tumors are bilateral in 20% of cases and are associated in 15–20% of cases with a synchronous endometrial carcinoma. Most of ECs are frequently associated with endometriosis or contain areas of endometrioid adenofibroma and endometrioid borderline tumor. Atypical endometriosis represents the precursor lesion of about 40% of ECs. Furthermore, the finding of a direct transition from atypical ovarian endometriosis to carcinoma is substantiated by the finding of common molecular alterations in both tumor and adjacent endometriosis [45,46,47].

### 4.1. Pathology of Endometrioid Carcinoma

Grossly, EC presents as a large mass of approximately 15–20 cm with smooth external surface. The tumor shows a cut surface characterized by solid-cystic and soft tissue associated with areas of hemorrhage. A residual endometriotic cyst may be identified at the periphery of the lesion.

**Figure 3:**
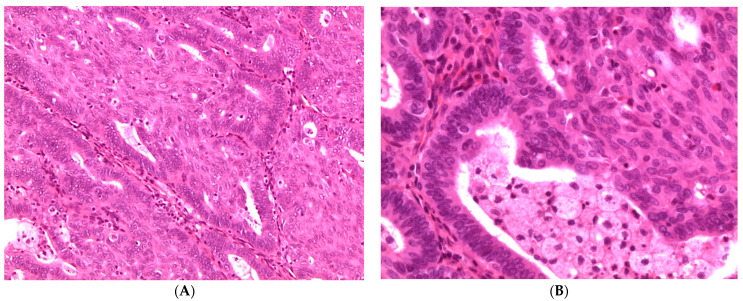
(**A**) and (**B**) Histologic appearance (hematoxylin and eosin, H&E) of low-grade endometrioid carcinoma with squamous differentiation (×100 and ×200, respectively).

Microscopically, EC are similar to uterine endometrial adenocarcinoma and its variants. Most ECs are low-grade carcinomas characterized by glandular, cribriform, and/or villoglandular patterns. The glands typically consist of tall stratified columnar cells with sparse eosinophilic cytoplasm. The cytoplasm may be focally mucinous, with mucin accumulating in the apical portion. Squamous differentiation is a hallmark of endometrioid neoplasms and represents a useful diagnostic feature (Figure 3). Mitotic count is approximately 5–10 mitoses per high power field.

High-grade ECs are poorly differentiated tumors characterized by solid growth, markedly atypical cells, and high mitotic activity. These tumors should be distinguished from SET variant of HGSC [48,49].

The so-called “confirmatory endometrioid features” are histologic characteristics reported in endometrioid neoplasms and include (i) metaplastic features (squamous, moral, hobnail, or mucinous) or other alterations in cellular phenotype (eosinophilic or secretory change); (ii) association with endometriosis, ovarian endometrioid adenofibroma or endometrioid borderline tumor; or (iii) presence of a synchronous uterine endometrioid neoplasm. The identification of these features provides strong support for a diagnosis of endometrioid carcinoma in morphologically equivocal cases [47].

The grading of ovarian EC is the same as for the uterine counterpart dividing tumors into grade 1 (tumors with less than 5% solid growth), grade 2 (tumors with 5–50% solid growth), and grade 3 (tumors show more than 50% solid growth), excluding areas of squamous differentiation.

Ovarian ECs have two different patterns of invasion: expansile and destructive. Expansile invasion is characterized by confluent glandular growth and has been correlated with low-stage and good prognosis. Destructive invasion shows neoplastic glands and small nests of tumor cells infiltrating the stroma associated with a marked desmoplastic reaction.

The immunohistochemical profile of EC includes diffuse positivity for PAX 8, Vimentin, Estrogen (ER), and Progesterone receptors (PR). Nuclear expression of β-catenin is present in a subset of ECs. Unlike HGSC, ECs are usually negative or only focal positive for WT1, p53, and p16. High-grade EC may show p53 mutation pattern staining [13].

### 4.2. Molecular Features of Endometrioid Carcinoma

The molecular landscape of ovarian EC is generally similar to its endometrial counterpart [50,51,52]. Analogous to the molecular subtypes of endometrial endometrioid carcinoma defined by The Cancer Genome Atlas (TCGA), four molecular classes of ovarian EC have been proposed: “ultramutated” due to *POLE* exonuclease domain mutations (~5%), “hypermutated” due to mismatch repair deficiency (MMRd)/microsatellite instability (MSI) (~13%), “*TP53*-mutated” (9–13%), and “no specific molecular profile” (NSMP; 69–73%). Furthermore, molecular alterations involve: the WNT/β-catenin signaling pathway (*CTNNB1* mutations, 53%), the PI3K pathway (*PIK3CA*, 40%; *PTEN*, 17%), the MAPK pathway (*KRAS*, 33%), and the SWI/SNF complex (*ARID1A*, 30%) [52]. Compared with endometrial carcinoma, ovarian EC has a similar frequency of β-catenin mutations but a lower rate of MSI and *PTEN* alterations.

The most common molecular abnormalities identified in ovarian ECs are somatic mutations of *CTNNB1*, the gene encoding β-catenin, and *PTEN*. *CTNNB1* mutations occur in 38–50% of cases and induce cytoplasmic and nuclear accumulation of β-catenin protein with subsequent participation in signal transduction and transcriptional activation through the formation of complexes with DNA-binding proteins. *CTNNB1* mutations are associated with low-grade tumors and favorable outcomes [53].

*PTEN* is a tumor suppressor gene located on chromosome 10q23.3 and is mutated in approximately 20% of ovarian EC. Somatic mutations of *PTEN* and loss of heterozygosity (LOH) at 10q23 frequently coexist and result in activation of the PI3K/AKT pathway that inhibits apoptosis. Another mechanism of triggering of *PIK3* signaling involves activating mutations in *PIK3CA*, which encodes the p110 catalytic subunit of PI3K. *PIK3CA* mutations in exons 9 and 20 have been identified in 20% of ovarian ECs and are associated with adverse prognostic factors [54].

Mutations in *ARID1A* (AT-rich interactive domain 1A gene), and loss of expression of the encoded protein BAF250a, occur in approximately 30% of EC but these are more frequent in CCC (50%). ARID1A is a component of a multiprotein chromatin-remodeling complex named SWI/SNF which enhances and represses transcription. It acts as a tumor suppressor gene [55]. Several studies have found *ARID1A* mutations and loss of expression of BAF250a in endometriosis adjacent to EC hypothesizing that this alteration may occur early in precursor lesions [47,52,56,57].

The reported frequency of MSI in ovarian ECs ranges from 10% to 20%. Similar to endometrial carcinoma, MSI has been demonstrated in patients with Lynch syndrome/hereditary non-polyposis colon cancer syndrome. These patients are characterized by an inherited germline mutation in the DNA mismatch repair (MMR) protein genes *MLH1*, *PMS2*, *MSH2*, or *MSH6* (“first hit”), but EC develops only after the deletion or mutation of the second corresponding allele (“second hit”). In sporadic tumors, MMR protein deficiency is frequently caused by inactivation of *MLH1* by promoter hypermethylation [58,59]. A recent study has identified clinically distinct EC subtypes: cases with *TP53* mutation demonstrate greater genomic complexity, are commonly FIGO stage III/IV at diagnosis (48%), are frequently incompletely debulked (44%) and demonstrate inferior survival; conversely, cases with *CTNNB1* mutation, which is mutually exclusive with *TP53* mutation, demonstrate low genomic complexity and excellent clinical outcome, and are predominantly stage I/II at diagnosis (89%) and completely resected (87%) [53]. Furthermore, subset of EC cases closely resembled HGSC, harboring *TP53* mutations, homologous recombination deficiency (HRD) mutation signatures and widespread copy-number variations [60].

## 5. Clear Cell Carcinoma

CCCs represent 10% of ovarian carcinomas and are most often low-stage at presentation accounting for approximately 25% of all FIGO stage I and II ovarian carcinomas [2]. Tumors are rarely bilateral. Similar to the endometrioid histotype, CCCs are strongly associated with endometriosis, while a portion of tumors contains adenofibromatous and borderline areas. Some studies have reported that tumors containing adenofibromatous component are associated with a more favorable prognosis than CCCs without adenofibromatous component (five-year survival 78.8% vs. 49.3%) [61]. Traditionally, CCCS is considered a high-grade malignancy, but stage I patients have a relatively favorable outcome with a five-year survival of 80–90% even in the presence of positive peritoneal cytology, ovarian capsule rupture, or capsular tumor involvement (stage IC). In contrast, advanced-stage tumors are associated with poor prognosis, even worse than that of a similar stage HGSC, and this is attributable to the low sensitivity of CCC to standard platinum-based chemotherapy. However, in some studies, MMR deficiency and/or Lynch syndrome-associated CCCs have been correlated to an unexpectedly long survival, and this may reflect tumor immunogenicity with the potential for immunotherapy in such cases [47,62].

### 5.1. Pathology of Clear Cell Carcinoma

Grossly, ovarian CCCs predominantly present as large unilateral masses, cystic, and solid in appearance, often containing foci of endometriosis and superficial adhesions.

**Figure 4:**
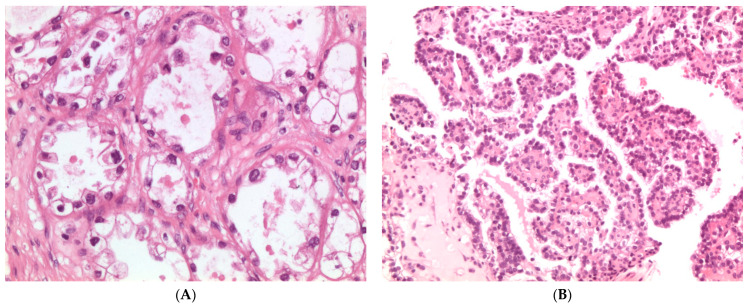
(**A**) Histologic appearance of clear cell carcinoma with tubulocystic pattern, tumor cells are polygonal to cuboidal and flattened with clear cytoplasm; (hematoxylin and eosin, H&E, ×200); (**B**) Papillary pattern shows small and regular papillae, frequently hyalinized (hematoxylin and eosin, H&E ×100).

Microscopically, CCCs exhibit a combination of a variety of patterns and cell types. Three classical architectural patterns are described: papillary, tubulocystic, and solid. Tumor cells show clear, eosinophilic, or flattened cytoplasm, and large, atypical nuclei with prominent nucleoli, but without significant pleomorphism. The atypia is therefore relatively uniform. Mitoses are usually less than 5/10 high power fields, less frequent than in other types of ovarian carcinomas. Remarkably, the presence of clear cytoplasm should not be used as the main diagnostic criterion of CCC since other ovarian tumor histotypes (i.e., EC and HGSC) may display clear cells. In addition, rare ovarian CCCs are composed entirely of eosinophilic cells. Detection of three characteristic microscopic features may help in the diagnosis of CCC: (1) multiple complex papillae; (2) densely hyaline basement membrane material or mucoid stroma expanding the cores of the papillae; and (3) hyaline bodies (Figure 4) [47,49].

CCC immunophenotype is specifically characterized by the expression of hepatocyte nuclear factor 1-beta (HNF-1β), negative staining for WT1, ER, and PR expression, and wild-type pattern of p53 expression [13].

### 5.2. Molecular Features of Clear Cell Carcinoma

The molecular abnormalities identified in ovarian CCCs are heterogeneous. Mutations in *ARID1A* gene have been demonstrated in approximately half of all ovarian CCC, as well as in areas of atypical endometriosis associated with these tumors. *PIK3CA* mutations occur in 30–40% of ovarian CCCs and commonly coexist with *ARID1A* alterations [63]. Interestingly, it has been suggested that the morphological features correlate with different molecular alterations. In particular, some studies report that *ARID1A* and *PIK3CA* mutations are more frequent in CCC endometriosis-associated, whereas they are less common in tumors with adenofibromatous component [56,57,64,65].

Among other alterations found in CCCs, *PTEN* mutations and/or loss of heterozygosity have been described in 5–20% of tumors. Interestingly, mutations in telomerase reverse transcriptase (*TERT*) promotor resulted in 15.9% ovarian CCC and may have a significant pathogenetic role in a subgroup of tumors [59]. Characteristically, unlike HGSC, CCCs are not associated with *BRCA* mutations; chromosomal instability and *TP53* mutations are usually absent. In contrast to endometrioid carcinoma, *CTNNB1* (β-catenin) alterations and MSI are uncommon in CCCs. However, some studies have found that a subset of ovarian CCCs, less than 10%, have germline mutations in genes encoding DNA MMR proteins and are associated with Lynch syndrome [66,67]. Therefore, ideally all ovarian CCCs should be tested by immunohistochemistry for MMR proteins or by MSI testing to detect those related to Lynch syndrome. Of note, MMR deficient/Lynch syndrome-associated tumors are unexpectedly correlated with good prognosis even in advanced stages [67].

## 6. Mucinous Carcinoma

In recent years, the number of ovarian mucinous tumors diagnosed as primary carcinomas has been largely reduced due to the classification of most ovarian mucinous tumors associated with pseudomyxoma peritonei as secondary neoplasms from the appendix and the recognition of metastatic adenocarcinomas, mainly of intestinal, pancreatic, and biliary tract origin, which mimic primary ovarian mucinous tumors.

Mucinous tumors represent 10–15% of all primary ovarian neoplasms and the vast majority (more than 80%) are benign or borderline tumors; mucinous carcinoma (MC) accounts for only 3–4% of ovarian carcinomas [2]. The origin of these tumors is unknown. Although a MC subgroup may derive from ovarian teratomas, in most cases no teratomatous component can be observed.

Features suggestive of primary ovarian MC include large size (>13 cm), unilaterality, and absence of ovarian surface involvement. In contrast, metastatic mucinous lesions are characteristically bilateral and smaller in size and they may be associated with pseudomyxoma peritonei.

### 6.1. Pathology of Mucinous Carcinoma

Grossly, MCs are usually large (8–40 cm; mean 15–20 cm in greatest dimension), unilateral, multilocular, or unilocular cystic masses containing mucinous fluid. They often exhibit papillary and solid areas that may be soft and mucoid or firm.

MCs are typically morphologically heterogeneous and may show admixture of benign, borderline, and carcinomatous components. Consequently, extensive sampling is required for microscopic examination. Most tumors exhibit gastrointestinal differentiation, or less frequently endocervical differentiation.

MCs may exhibit two patterns of growth: (1) an expansile type without obvious stromal invasion but with back-to-back or complex malignant glands with minimal or absent stroma; and (2) an infiltrative type, with obvious stromal invasion and frequently associated with a desmoplastic stromal reaction. The expansile growth pattern is associated with a more favorable prognosis than the infiltrative pattern [68]. Rarely, mural nodules of anaplastic carcinoma or even high-grade sarcoma may be present in the context of MC [69].

MC immunoprofile is characterized by diffuse and strong positivity for CK7 with variable, negative to irregular, but generally not diffuse expression of CK20, although teratoma-associated mucinous tumors are often CK7 negative/CK20 positive. All of the primary tumors that are not associated with cystic teratomas are negative for SATB2. CDX2 is usually expressed, whereas WT1, ER, and PR expression is absent, unlike endometrioid (ER+) and serous (ER+ and WT1+) carcinomas.

### 6.2. Molecular Features of Mucinous Carcinoma

The molecular profile of MCs differs from other histotypes of ovarian carcinoma. The most common molecular alterations are copy-number loss of *CDKN2A* (76%) followed by mutations in *KRAS* and *TP53* (both 64%). *HER2* amplification (26% of cases) and mutations in *RNF43*, *BRAF*, *PIK3CA*, and *ARID1A* (8–12% of cases) were the next most frequent [70]. Some authors generated a model of progression from benign to borderline to localized low-grade MC and progressively through to high-grade tumors. *KRAS* or *CDKN2A* alterations have been identified in precursor lesions and therefore are considered to be early events [71,72]. Mucinous borderline tumors are significantly more likely to have both events and may have additional copy-number alterations. Low-grade MC have yet more copy-number alterations and are more likely to have a *TP53* mutation. Copy-number alterations are key drivers associated with increasing grade and metastatic progression and are potential prognostic markers. Of note, *KRAS* mutations are almost mutually exclusive of *HER2* amplification. Approximately 34% of MCs have neither *HER2* amplification nor *KRAS* mutation, and these cases are associated with an increased risk of recurrence and poor prognosis compared with tumors with either molecular alterations.

The most important prognostic parameter is FIGO stage, in fact stage I tumors have an excellent prognosis, while prognosis is poor in cases with extraovarian spread.

## 7. Conclusions

Traditional and molecular pathology improves ovarian tumor classification in a fashion that will allow us to focus on categories that more effectively convey information about predicted behavior and response to therapy. The need for an accurate diagnosis is particularly essential in the current era of personalized therapy for a successful specific treatment.

Nowadays, the integration of histopathological and molecular data is even more important for the discussion of the clinical management of patients within interdisciplinary groups (Molecular Tumor Board) in which oncological, molecular biology, bioinformatics, pathology, clinical pharmacology, and genetic counseling expertise are involved.

## Figures and Tables

**Table 1:** Molecular and pathologic classification of ovarian carcinomas.

	High-Grade Serous Carcinoma	Low-Grade Serous Carcinoma	Endometrioid Carcinoma	Clear Cell Carcinoma	Mucinous Carcinoma
Percentage of all ovarian carcinomas	70%	<5%	10%	6–10%	3–4%
Site of origin	Fallopian tube	Endosalpingiosis/ Fallopian tube	Endometriosis	Endometriosis	Teratoma/ Unknown
Precursor lesion	Serous tubal intraepithelial carcinoma (STIC)	Serous borderline tumor	Atypical endometriosis; endometrioid borderline tumor	Atypical endometriosis; clear cell borderline tumor	Mucinous borderline tumor
Hereditary cancer syndrome	BRCA1/2-associated hereditary breast and ovarian cancer syndrome (HBOC)	-	Lynch syndrome	Lynch syndrome	-
Molecular alterations	*TP53* *BRCA1/2* HRD Chromosomal instability Copy-number alterations	*KRAS* *NRAS* *BRAF* *HER2*	*CTNNB1* *PIK3CA* *PTEN* *KRAS* *ARID1A* *MSI* *POLE* *TP53*	*ARID1A* *PIK3CA* *PTEN* *MSI*	*CDKN2A* copy-number loss *KRAS* *HER2* amplification *TP53*
Potential molecular targeted therapies	PARP inhibitors; Immune checkpoint inhibitors	MEK inhibitor	mTOR inhibitors; Immune checkpoint inhibitors	Tyrosine kinase inhibitor; Immune checkpoint inhibitors	Trastuzumab

**Table 2:** Criteria for assigning primary site in high-grade serous carcinoma.

Primary Site	Criteria for Diagnosis
Fallopian tube	Presence of: STIC or invasive HGSC in fallopian tube or Part or entire length of tube inseparable from adnexal mass
Ovary	Both fallopian tubes separate from ovarian mass and No STIC or mucosal HGSC in either tube
Tubo-ovarian	Fallopian tubes and ovaries not available for complete examination and Pathological findings consistent with extrauterine HGSC
Peritoneal	Both tubes and both ovaries fully examined and No gross or microscopic evidence of STIC or HGSC in tubes or ovaries
